# High‐Throughput Manufacturing of Multimodal Epidermal Mechanosensors with Superior Detectability Enabled by a Continuous Microcracking Strategy

**DOI:** 10.1002/advs.202305777

**Published:** 2023-11-30

**Authors:** Jianing An, Van Thai Tran, Hai Xu, Wenshuai Ma, Xingkuan Chen, Truong‐Son Dinh Le, Hejun Du, Gengzhi Sun, Young‐Jin Kim

**Affiliations:** ^1^ Institute of Photonics Technology Jinan University Guangzhou 510632 P. R. China; ^2^ Singapore Centre for 3D Printing Nanyang Technological University 50 Nanyang Avenue Singapore 639798 Singapore; ^3^ College of Materials Science and Technology Nanjing University of Aeronautics and Astronautics Nanjing 211100 P. R. China; ^4^ Department of Chemistry Jinan University Guangzhou 510632 P. R. China; ^5^ Department of Mechanical Engineering Korea Advanced Institute of Science and Technology (KAIST) Daejeon 34141 Republic of Korea; ^6^ Institute of Advanced Materials (IAM) Nanjing Tech University (NanjingTech) Nanjing 211816 P. R. China

**Keywords:** continuous microcracking, high‐throughput laser manufacturing, multimodal epidermal mechanosensors, rigid‐soft hybrid sensing layer, superior detectability

## Abstract

Non‐invasive human‐machine interactions (HMIs) are expected to be promoted by epidermal tactile receptive devices that can accurately perceive human activities. In reality, however, the HMI efficiency is limited by the unsatisfactory perception capability of mechanosensors and the complicated techniques for device fabrication and integration. Herein, a paradigm is presented for high‐throughput fabrication of multimodal epidermal mechanosensors based on a sequential “femtosecond laser patterning‐elastomer infiltration‐physical transfer” process. The resilient mechanosensor features a unique hybrid sensing layer of rigid cellular graphitic flakes (CGF)‐soft elastomer. The continuous microcracking of CGF under strain enables a sharp reduction in conductive pathways, while the soft elastomer within the framework sustains mechanical robustness of the structure. As a result, the mechanosensor achieves an ultrahigh sensitivity in a broad strain range (GF of 371.4 in the first linear range of 0–50%, and maximum GF of 8922.6 in the range of 61–70%), a low detection limit (0.01%), and a fast response/recovery behavior (2.6/2.1 ms). The device also exhibits excellent sensing performances to multimodal mechanical stimuli, enabling high‐fidelity monitoring of full‐range human motions. As proof‐of‐concept demonstrations, multi‐pixel mechanosensor arrays are constructed and implemented in a robot hand controlling system and a security system, providing a platform toward efficient HMIs.

## Graphical abstract

A multimodal epidermal mechanosensor with superior detectability is achieved by a high‐throughput “femtosecond laser patterning‐elastomer infiltration‐physical transfer” manufacturing method. The continuous microcracking of the rigid conductive frameworks results in ultrahigh strain sensitivities, while the soft elastomer filler renders the device with a wide sustainable strain range. The multimodal mechanosensor can acquire diverse physiological and physical signals in a high‐fidelity fashion.

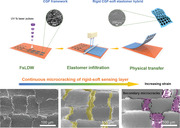

## 1 Introduction

Intelligent biomimicry of the human somatosensory system through electronics drives the innovation in human‐machine interactions (HMIs) so as to bring a new frontier for artificial intelligence, machine learning, and internet of things.^[^ ^1^ ^]^ Epidermal mechanosensors enable a shortcut to accomplish the seamless communication between humans and machines by means of precisely acquiring the information of human activities and rapidly transducing the tactile stimuli into electrical signals for subsequent data encoding.^[^ ^2^ ^]^ To work collaboratively with the soft and curvilinear human body thus implementing the complex sensations, it is crucial to endow the epidermal sensory system with soft, resilient, deformable, and lightweight features in order to form an intimate interface with human skin.^[^ ^3^ ^]^ Another stringent requirement for the mechanosensors is to detect multimodal external forces with the capability of retaining a high sensitivity in a wide sustainable strain range.^[^ ^4^ ^]^ In addition, the facile fabrication and assembly of individual mechanosensors into patterned arrays should also be concerned, so as to quantify the tactile signal distribution with high spatial resolution.^[^ ^5^ ^]^ Hence, the design of novel epidermal mechanosensors that encompass all of the stated attributes and the development of high‐throughput sensor array construction method are in imminent demand.

To date, tactile sensing has been extensively implemented based on various transduction mechanisms, such as piezoresistive, piezocapacitive, piezoelectric, and triboelectric effects.^[^ ^4a^ ^]^ In particular, the piezoresistive mechanosensors featuring low energy consumption, static/dynamic bi‐functional sensing capability, and simple device structure are widespread.^[^ ^6^ ^]^ To achieve piezoresistive mechanosensors with combined mechanical compliance and excellent sensing performances, elastomers are widely adopted as the supporting blocks because of their intrinsic softness with Young's modulus of 1–100 kPa, which is comparable to that of human skin (typically in the range of 20–200 kPa).^[^ ^3^ ^]^ Conductive materials such as metallic nanostructures, conductive polymers, and carbonaceous nanomaterials can be deposited onto or embedded into the elastomer substrates, or blended with the elastomers for device construction.^[^ ^7^ ^]^ However, the sensitivity and the detection range are mutually contradictive for conventional piezoresistive strain sensors, because a high sensitivity originates from a sharp decrease of the conductive pathways under a subtle strain, whereas a wide detection range requires a sustained electron transport trajectory even when largely stretched.^[^ ^8^ ^]^ In order to obtain a superior detectability for full‐range human motions, numerous efforts have been paid to simultaneously achieve a high sensitivity (GF > 100) and a wide detection range (>50%) for a strain sensor.^[^ ^7^, ^9^ ^]^ Crack engineering provides an effective way to boost high sensing performances of piezoresistive strain sensors, for instance, many parallel crack‐based sensors have demonstrated high strain sensitivity and low detection limit, profiting from the significant decrease of conductive pathways enabled by the crack disconnection and the generation of tunneling channels.^[^ ^10^ ^]^ Nevertheless, parallel crack‐based mechanosensors normally suffer from narrow detection range (<5% strain) and limited sensing capability for tension. Although the detection range can be widened by generating network microcracks to the sensing layer, the sensitivity at low strain range is typically compromised.^[^ ^9a^ ^]^ In addition, the weak interfacial adhesion between mechanically mismatched sensing layer and elastomer substrate raises severe durability issues.^[^ ^11^ ^]^ Embedding conductive fillers in elastomer hosts is an alternative approach to fabricate mechanosensors toward multimodal mechanical stimuli, such as tension, compression, and torsion, where the conductive pathways are established through the percolation networks.^[^ ^12^ ^]^ However, a high volumetric percentage of conductive fillers in the blended materials inevitably reduces the stretchability of the composites. Furthermore, the stacking and aggregation of low‐dimensional nanostructures normally suppresses the efficient electron transport, leading to compromised sensitivity and sluggish response/recovery speeds.^[^ ^7c^ ^]^ To alleviate these problems, 3D conductive cellular architectures have been employed to create foam‐shaped multimodal mechanosensors.^[^ ^13^ ^]^ The regular cellular structures assembled from 2D nanomaterials (e.g., graphene) by foam‐templating or freeze‐casting methods provide interconnected conductive networks; then soft elastomers are infiltrated into the pore space to ensure the mechanical resilience and robustness of the composite.^[^ ^14^ ^]^ However, the obtained bulky mechanosensors (thickness of the sensing layer is in millimeter magnitude) typically exhibit limited stretchability, as well as mediocre sensitivity to subtle strain, because tiny strain‐induced resistance change is less pronounced for thick foam with abundant conductive pathways.^[^ ^15^ ^]^ Besides, the slippage of overlaps/folds formed inter/intra flexible graphene nanosheets in the assembled foam structure further suppress the effective resistance change under subtle strain.^[^ ^7c^ ^]^ In another aspect, the scalable production of mechanosensor arrays to realize high‐spatial‐resolution multiplexed tactile sensing remain challenging. The state‐of‐the‐art methods for fabricating mechanosensor array generally rely on the preparation of individual sensing elements and the subsequent manual alignment of pixels into a matrix, which is time‐consuming and difficult to scale up.^[^ ^13b^ ^]^

Herein, we present a high‐throughput manufacturing strategy based on a sequential “UV femtosecond laser patterning‐elastomer infiltration‐physical transfer” process to produce ultrasensitive, mechanically resilient, and multimodal mechanosensor arrays featuring a thin rigid‐soft hybrid sensing layer. The high sensitivity in a wide sustainable strain range (GF of 371.4 in the first linear range of 0–50%, and maximum GF of 8922.6 in the range of 61–70%), low detection limit (0.01%), and multimodal sensing capabilities of the as‐fabricated mechanosensors are fully characterized. The underlying mechanisms for such a superior detectability are highlighted, where the continuous microcracking of the laser‐induced cellular graphitic flakes (CGF) in the conductive framework plays an essential role for the sharp reduction in conductive pathways, while the infiltrated soft elastomer is responsible for the softness, mechanical robustness and resilience of the device. As the consequences, our mechanosensors achieve high‐fidelity monitoring of diverse vital physiological and physical signals from the human body, including the wrist pulse signal, the frowning/swallowing motion, and the finger/wrist/elbow flexion. Moreover, as proof‐of‐concept examples, multi‐pixel mechanosensor arrays that can differentiate the multi‐point pressure distribution and record the motion trajectory in real time are arbitrarily designed and implemented in a robot hand controlling system and a security system. It is believed that the high‐throughput manufacturing strategy proposed in this study paves the way for intelligent HMIs based on epidermal electronics.

## 2 Results and Discussion

**Figure 1:**
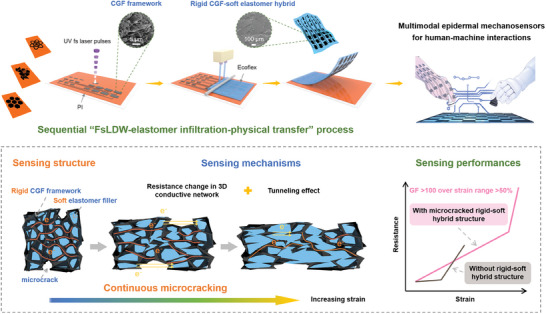
Conceptual illustration of the design principle and the manufacturing strategy. The multimodal epidermal mechanosensor arrays can be constructed through a sequential “FsLDW‐elastomer infiltration‐physical transfer” process. The mechanosensor features a unique rigid CGF‐soft elastomer hybrid structure that enables continuous microcracking under an increasing strain, so as to achieve a high strain sensitivity over a wide detection range. The soft and stretchable mechanosensor array can be conformably attached to human body to detect various physical and physiological signals for intuitive HMI systems.

**Figure** **1** schematically depicts the feasible construction of epidermal multimodal mechanosensor array by virtue of a high‐throughput manufacturing strategy. To achieve a combined high strain sensitivity of crack‐based sensors and a wide detection range of foam‐based sensors, the device fabrication process begins with the programmable synthesis of a thin‐layer CGF by UV femtosecond laser direct writing (FsLDW) using polyimide (PI) as the precursor. Extensive studies have shown that laser‐induced pyrolysis and graphitization can effectively convert thermoplastic polymers to porous graphene structures, which is promising for mass‐production of graphene electronics.^[^ ^16^ ^]^ However, the widely adopted CO_2_ laser with a wavelength of 10.6 µm generally requires a high input power (e.g., several watts) to elevate the processing temperature for graphitization of the precursor due to the poor absorption of infrared (IR) light by PI.^[^ ^17^ ^]^ The significant photothermal effect adversely results in buckling of the patterned film, delamination of graphene layer from PI, and/or ablation of the polymer substrate, posing severe problems for scalable and reliable construction of epidermal mechanosensor arrays.^[^ ^18^ ^]^ To tackle these issues, a UV femtosecond laser is employed as the energy source in this work, which enables a fundamentally different laser‐material interaction mechanism compared to other IR lasers.^[^ ^19^ ^]^ First of all, the high photon energy of UV irradiation can trigger direct atomic bond breaking in PI through a photochemical process, which facilitates the transformation of sp^3^‐bonding to sp^2^‐bonding carbon atoms.^[^ ^18^ ^]^ Besides, the ultrashort and high‐repetition‐rate femtosecond laser pulses allow for the optimal heat accumulation in a small volume for efficient graphitization of the dissociated polymer molecules, providing a low‐temperature processing route with minimal thermal relaxation to surroundings.^[^ ^19^, ^20^ ^]^ Furthermore, PI exhibits a much stronger absorption of UV light compared to IR light, so as to enable a more focused laser spot thus achieving a decreased penetration depth and a higher spatial resolution.^[^ ^18^ ^]^ The thin‐layer CGF synthesized by UV laser irradiation serves as the rigid conductive framework for subsequent soft elastomer infiltration and physical transfer, producing a free‐standing rigid‐soft hybrid sensing layer with abundant microcracks. Attributed to the rigid‐soft hybrid nature and the continuous microcracking of CGF under incremental strains, the CGF‐Ecoflex sensor possesses superior detectability, softness, and stretchability, enabling its intimate contact with human skin and the capability of monitoring external deformations. The proposed sequential “laser patterning‐elastomer infiltration‐physical transfer” process allows for scalable production of mechanosensor arrays, which can be conformably attached to human body to acquire a rich variety of physical and physiological signals in a high‐fidelity fashion, thereby promoting their utilization in diverse intelligent HMI systems.

**Figure 2:**
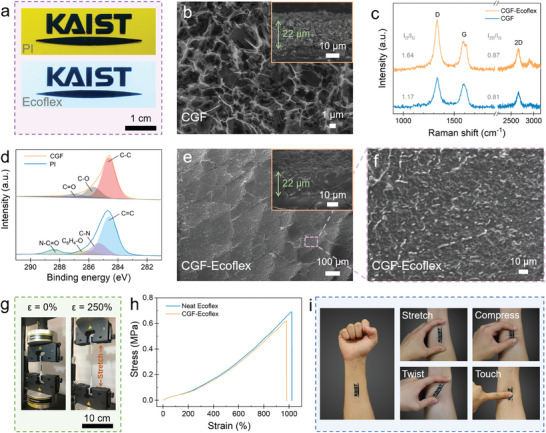
Preparation and characterizations of the CGF‐Ecoflex hybrid film. a) Photographs of a representative “KAIST” logo patterned on PI sheet and transferred to Ecoflex. b) SEM image of CGF with porous structures. Inset: cross‐sectional view of the CGF layer. c) Raman spectra of CGF and CGF‐Ecoflex. d) High‐resolution XPS C1s spectra of pristine PI and CGF. e) SEM image of CGF‐Ecoflex with abundant microcracks. Cross‐sectional view (inset of e) and enlarged view f) show the full infiltration of Ecoflex within the CGF framework. g) Photographs of the CGF‐Ecoflex sample at the beginning and during the uniaxial tensile test. h) Stress–strain curves of neat Ecoflex and CGF‐Ecoflex. i) Photographs showing the CGF‐Ecoflex hybrid film can be conformably laminated onto human forearm and bear diverse mechanical deformations.

The FsLDW enables programmable synthesis of CGF from the carbon‐rich PI precursor in an arbitrary patterning fashion. The top panel in **Figure** **2**a shows an artistic CGF pattern obtained with a UV femtosecond laser power of 400 mW and a fast laser writing speed of 100 mm s^−1^; other arbitrary patterns are presented in Figure S1 (Supporting Information). The scanning electron microscopy (SEM) image of CGF (Figure 2b) clearly reveals a foam‐like structure with pore size of a few micrometers, as a result of the rapid gas release during polymer decomposition.^[^ ^16a^ ^]^ The thickness of the porous layer is ≈22 µm as shown in the cross‐sectional SEM image (Figure 2b inset). Its Raman spectrum in Figure 2c displays the typical graphitic feature with distinctive D band at 1334 cm^−1^, G band at 1595 cm^−1^, and 2D band at 2667 cm^−1^, respectively.^[^ ^16^, ^21^ ^]^ The intense and symmetric 2D profile with the *I*_2D_/*I*_G_ ratio of 0.81, together with the high *I*_D_/*I*_G_ ratio of 1.17, indicates CGF has a few‐layer graphene structure with disordered fringes.^[^ ^22^ ^]^ The CGF possesses superior electrical conductivity, registering an average sheet resistance (*R*_s_) of ≈42.2 Ω sq^−1^ as measured by a four‐point probe method (Figure S2, Supporting Information). The elemental composition of CGF was characterized by X‐ray photoelectron spectroscopy (XPS). Quantification analysis based on XPS survey scan (Figure S3, Supporting Information) shows a high carbon content of 96.9 at.% and low contents of oxygen (2 at.%) and nitrogen (1.1 at.%), suggesting an almost full conversion of CGF.^[^ ^18^ ^]^ In the high‐resolution C1s spectrum of CGF (top panel in Figure 2d), three deconvolved peaks are observed, corresponding to C─C (284.6 eV), C─O (285.7 eV) and C═O (286.9 eV) bonds respectively.^[^ ^23^ ^]^ As a comparison, the C1s spectrum of pristine PI (bottom panel in Figure 2d) is composed of C═C (284.5 eV), C─N (285.3 eV), C_6_H_4_─O (286.2 eV), and N─C═O (288.4 eV) species.^[^ ^24^ ^]^ These indicate that C─N, C─O, and C═O bonds were broken under UV laser irradiation, while the aromatic and imidic rings in PI were rearranged to form sp^2^ carbon structures.^[^ ^23^ ^]^ It is noteworthy that the microstructures and conductivity of CGF can be finely tuned by laser processing parameters. As shown in Figures S2 and S4 (Supporting Information), a silvery film composed of sheet‐like graphitic structures was obtained with a laser power of 200 mW. The relatively high sheet resistance and weak 2D peak in Raman spectrum indicate a lower graphitization degree of the product due to insufficient reaction temperature.^[^ ^25^ ^]^ Although highly graphitized porous structures were achieved at a higher laser power, the sheet resistance was slightly increased as a result of oxidization.^[^ ^25^ ^]^ Further increasing the laser power to 600 mW caused partial CGF ablation/delamination owing to heat accumulation.^[^ ^25^ ^]^ Therefore, the laser power was optimized to 400 mW to synthesize high‐quality CGF structures. The foam‐like structure of CGF is favorable for elastomer infiltration, obtaining a rigid CGF‐soft Ecoflex hybrid layer. SEM observations (top and cross‐sectional views in Figure 2e) reveal the full embedment of Ecoflex in CGF framework throughout the entire thickness, forming a good interface between CGF and Ecoflex (Figure 2f) thus enabling the successful transfer of complex CGF patterns onto elastomeric substrates (bottom panels in Figure 2a; Figure S1, Supporting Information). In addition, numerous microcracks were generated to release strains induced by physical transfer.^[^ ^10^ ^]^ The Raman spectrum of CGF‐Ecoflex hybrid (Figure 2c), in comparison with that of CGF, suggests the preservation of graphitic structure in the hybrid film with a similar *I*_2D_/*I*_G_ ratio. The slight downshift of D, G, and 2D peak positions can be attributed to the local strain induced by elastomer infiltration within the CGF framework,^[^ ^21b^ ^]^ while the increased *I*_D_/*I*_G_ ratio and the presence of D+G band at 2905 cm^−1^ (a disorder‐induced mode) are ascribed to the wrinkles and defects.^[^ ^20^, ^26^ ^]^ The electrical and mechanical properties of the CGF‐Ecoflex hybrid were probed subsequently. A drastic increase in *R*_s_ to a high range of 500–2000 Ω sq^−1^ is observed for CGF‐Ecoflex hybrid films, which arises from the generated microcracks. The tensile stress–strain curves of neat Ecoflex and CGF‐Ecoflex hybrid samples are compared in Figure 2h. The CGF‐Ecoflex hybrid exhibits a skin‐matching Young's modulus of 61 kPa and a superior stretchability with an elongation at break of 980%, which are analogous to those of neat Ecoflex. Furthermore, the rigid CGF‐soft Ecoflex hybrid also maintained its structural integrity under a compressive force (Figure S5a,b, Supporting Information) and exhibited high resistance to a sticky tape peeling test (Figure S6, Supporting Information). By contrast, the rigid CGF structures collapsed during the compression test (Figure S5c,d, Supporting Information); and a large amount of CGF material was removed from the PI sheet by sticky tape, resulting from the weak adhesion between CGF layer and PI sheet (Figure S6, Supporting Information). Hence, the softness, stretchability, and mechanical robustness of the rigid‐soft hybrid film enable its compliant contact with human skin and the accommodation of diverse deformations (Figure 2i), so as to be implemented in various on‐skin electronic applications.

**Figure 3:**
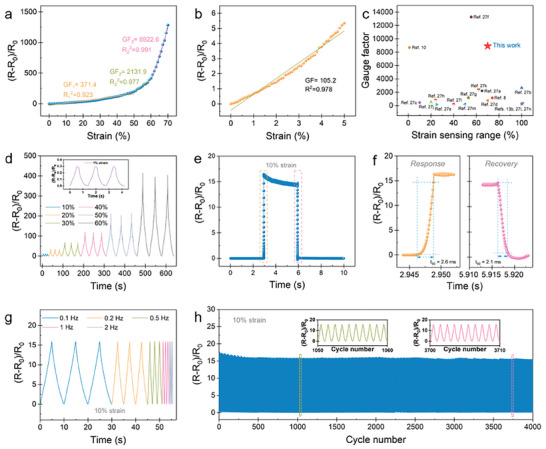
Tensile strain sensing performances. a) Relative resistance variation of the sensor as a function of tensile strain. b) The sensitivity of the sensor in the minute strain range of 0–5%. c) Comparison of strain sensing performances of our sensor to other recently reported works. d) Dynamic resistance responses of the sensor to different strain levels. Inset: responses to cyclic loading of a subtle strain of 1%. e) Real‐time response to a strain of 10%. f) Corresponding response and recovery times extracted from (e). g) Dynamic resistance responses of the sensor as a function of frequency at a strain of 10%. h) Durability test under a repetitive strain of 15% for 4000 cycles.

To demonstrate its multimodal mechanosensing capability, the CGF‐Ecoflex hybrid film was assembled into a piezoresistive device; the resistance of the as‐assembled mechanosensor was ≈807 Ω, as determined by the current–voltage (*I*–*V*) plot for the device under 0% strain (Figure S7, Supporting Information). The strain sensing performances were first evaluated. **Figure** **3**a plots the relative resistance change (∆*R*/*R*_0_, ∆*R* = *R*−*R*_0_) as a function of the applied tensile strain (*ε*); a nearly linear dependence of ∆*R*/*R*_0_ on strain is shown in a wide strain range of 0–50%, with a high gauge factor (GF) of 371.4 where GF = (∆*R*/*R*_0_)/*ε*. The GF increases to 2131.9 in the strain range of 50–60%, finally rises to an ultrahigh value of 8922.6 in the strain range of 60–70%. The sensor also exhibited a high GF of 105.2 in the small strain range of 0–5% (Figure 3b). The Ashby diagram of sensitivity and detection range of the strain sensor in Figure 3c shows that our CGF‐Ecoflex hybrid sensor possesses combined high sensitivity of crack‐based sensors and wide detection range of foam‐based sensors, which are comparable to or outperform those of recently reported strain sensors.^[^ ^8^, ^10^, ^13^, ^27^ ^]^ The dynamic resistance responses of the CGF‐Ecoflex hybrid sensor to different strain levels are presented in Figure 3d (bias voltage = 1 V), showing stable and reversible behaviors during stretching‐releasing cycles (at a sweep rate of 0.1 mm s^−1^) in a wide strain range. Notably, reliable responsive signals can be detected to a subtle strain of 0.01% (Figure S8, Supporting Information), implying an ultralow detection limit of the strain sensor. The hysteresis effects of the sensor during different levels of strain loading‐unloading cycles are shown in Figure S9 (Supporting Information). Low hysteresis is exhibited at each strain level, which may arise from the viscoelasticity of Ecoflex and the CGF‐Ecoflex interfacial interactions.^[^ ^28^ ^]^ The real‐time resistance response of the sensor to a strain of 10% is shown in Figure 3e; the enlarged views of stretching and releasing processes reveal its ultrafast response and recovery times of 2.6 and 2.1 ms, respectively (Figure 3f), where the response and recovery times were defined as the time required for 90% of the total resistance change in a dynamic cycle. In addition, the strain sensor displays a frequency‐independent responsive characteristic when sensing a strain of 10% within the tested frequency range of 0.1–2 Hz (Figure 3g). The long‐term stability was assessed by repetitive strain (10%, 30%, and 50%) loading‐unloading at a frequency of 1 Hz. As shown in Figure 3h and Figure S10 (Supporting Information), the viscoelasticity of Ecoflex caused signal overshoot in the incipient few hundred cycles; then negligible signal degradation is observed in the following (up to 4000) cycles, demonstrating the excellent mechanical durability of the strain sensor.

**Figure 4:**
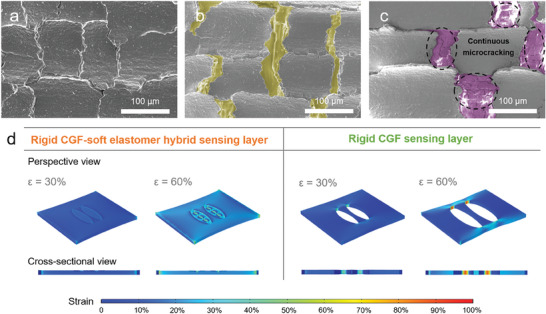
Strain sensing mechanisms of the CGF‐Ecoflex hybrid mechanosensor. SEM images of the CGF‐Ecoflex hybrid thin layer a) in its original status, b) under a tensile strain of 30%, and c) under a tensile strain of 60%, respectively. The widening of microcracks under tensile strains is in pseudo color in (b) and (c), the continuous microcracking phenomenon is circled in (c). d) FEA results show the strain distribution around the microcracks under different tensile strains for a rigid‐soft hybrid sensing layer (left) versus a rigid sensing layer (right).

The exotic strain sensing characteristics can be attributed to the continuous microcracking of the rigid CGF in the foam‐shaped rigid‐soft hybrid sensing layer. To elucidate the sensing mechanism, the microstructures of CGF‐Ecoflex hybrid sensor under increasing strain were investigated using SEM (**Figure** **4**a–c) and in situ optical microscope (Figure S11, Supporting Information). As shown in Figure 4a, the microcracks generated during the transfer process divided the CGF‐Ecoflex hybrid sensing layer into isolated islands, with microcrack edges bridging adjacent islands. Upon loading tensile strain to the sensor, the interconnected graphitic flakes in the 3D conductive framework are separated and microcracks are widened (Figure 4b), resulting in a reduction in the conductive pathways. For tensile strain in a small range (0–50%), the sensing behavior is prominently governed by the contact resistance variation originating from the separation/re‐contact of graphitic flakes and opening/closure of microcracks.^[^ ^10^, ^29^ ^]^ As shown in Figure S7 (Supporting Information), linear *I*–*V* characteristics are observed for the device subjected to strains up to 50%, implying an Ohmic conduction for the electron transport.^[^ ^10^ ^]^ At a higher strain level (60–70%), the adjacent graphitic flakes and neighbouring microcracks lose direct electrical contact (Figure 4c), therefore the tunneling effect dominates the sensing principle.^[^ ^30^ ^]^ The *I*–*V* curve of the sensor at 70% strain is nonlinear (Figure S12a, Supporting Information), while the linear relationship between ln (*I*/*V* ^2^) and 1/*V* is obtained in the voltage range of 0.5–1 V (Figure S12b, Supporting Information), indicating the electron conduction follows a Fowler–Nordheim tunneling mechanism.^[^ ^31^ ^]^ It is noteworthy that the soft Ecoflex in the foam‐shaped hybrid layer played an essential role in absorbing the mechanical energy when the sensor was subjected to large tensile strains.^[^ ^32^ ^]^ As highlighted in Figure 4c, continuous microcracking of the rigid CGF framework under an increasing strain is observed, which prevented the structural fracture by confining the elongation of microcracks.^[^ ^32a^ ^]^ The influence of the sensing layer composition on the microcrack evolution was supported by the finite element analysis (FEA). As shown in Figure 4d, the strain around the microcracks of a rigid‐soft hybrid sensing layer was higher and distributed in a wider area compared to that of a rigid sensing layer under the same tensile strain (ε = 30%). When the rigid‐soft hybrid sensing layer was further stretched to ε = 60%, the localized strain was undertaken by the soft component and released by generating secondary microcracks, thus avoiding strain concentration (left panel in Figure 4d). For comparison, the strain tended to concentrate on the two ends of microcracks for a rigid sensing layer under 60% tensile strain, which caused the microcrack elongation in the direction perpendicular to the stretching and the breakage of conductive pathways (right panel in Figure 4d). As a proven, polydimethylsiloxane (PDMS) was employed as the polymeric filler to be infiltrated into CGF framework. As shown in Figure S13a–c (Supporting Information), the PDMS is not fully embedded within CGF network, leaving plenty of voids in the hybrid structure. Consequently, the strain sensor based on CGF‐PDMS hybrid exhibited inferior GFs and a narrower sensing range (GF = 38.9 for strain range of 0–11%, and GF = 2077.6 for strain range of 12–23%), as plotted in Figure S13d (Supporting Information).

**Figure 5:**
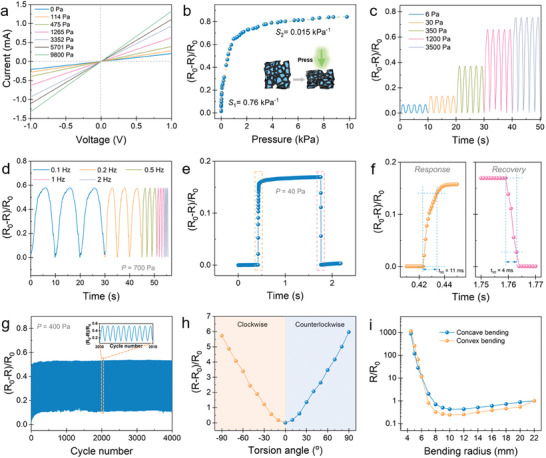
Sensing performances of the CGF‐Ecoflex hybrid mechanosensor to pressure, torsion, and bending. a) *I*–*V* curves of the device under different pressures. b) Relative resistance variation of the sensor as a function of pressure. Inset: schematic illustration of pressure sensing mechanism. c) Dynamic resistance responses of the sensor to different pressures at a frequency of 0.5 Hz. d) Dynamic resistance responses of the sensor to a pressure of 700 Pa at different frequencies. e) Real‐time response to a pressure of 40 Pa. f) Corresponding response and recovery times extracted from (e). g) Durability test under a repetitive pressure of 400 Pa for 4000 cycles. h) Plots of ∆*R*/*R*_0_ as a function of torsion angle in both clockwise and counterclockwise directions. (i) Resistance responses to concave and convex bending deformations.

Subsequently, pressure sensing test ensued by applying different static loadings on the device while monitoring its resistance variation. **Figure** **5**a displays the *I*–*V* curves of the CGF‐Ecoflex hybrid sensor from −1 to 1 V under pressures in the range of 0–10 kPa. The current shows a linear relationship to voltage obeying Ohm's law and the device resistance decreases with the augment of pressure. The relative resistance variation (∆*R*/*R*_0_, where ∆*R* = *R*_0_−*R*, *R*_0_ and *R* respectively represent the initial resistance and the resistance under pressure) is plotted as a function of pressure (*P*) in Figure 5b, two linear regions are observed from the curve. The pressure sensitivity (*S*) of the device, defined as *S* = *δ*(∆*R*/*R*_0_)/*δP*,^[^ ^33^ ^]^ is 0.76 kPa^−1^ in the low pressure region (0–1 kPa) and 0.015 kPa^−1^ in the high pressure region (up to 10 kPa). The pressure sensing mechanism is schematically depicted in Figure 5b inset. The pressure on the CGF‐Ecoflex hybrid induces a structural change of the CGF network, so that the interconnection of neighboring struts/cell walls transforms from “point‐to‐point” mode to “point‐to‐face” and “face‐to‐face” modes, resulting in more graphitic conductive pathways in the film.^[^ ^13^, ^34^ ^]^ The resistance variation slows down at a higher pressure, because close surface contacts are already established between struts/cell walls.^[^ ^35^ ^]^ Along with the high sensitivity, the sensor also exhibited a low detection limit of 0.14 Pa, as resolved by loading a piece of weighing paper to the sensor (Figure S14, Supporting Information). Dynamic pressure loading–unloading tests were carried out at a bias voltage of 1 V; the sensor exhibited steady cyclic responses and a good adaptability to wide‐range pressures (Figure 5c) and different frequencies (Figure 5d). A rapid response‐recovery behavior of the device is observed from a pressure loading‐unloading process (Figure 5e); the response and recovery times to the pressure of 40 Pa are determined to be 11 and 4 ms, respectively (Figure 5f). The long‐term cycling stability of the pressure sensor was examined under a repetitive pressure of 400 Pa (Figure 5g), the resistance response (∆*R*/*R*_0_) gradually diminishes during the first 200 cycles which may arise from the viscoelasticity of the Ecoflex, and then becomes stable in the following (up to 4000) cycles, suggesting its remarkable mechanical durability. In addition, the CGF‐Ecoflex hybrid sensor showed excellent sensing performances to torsion and bending as well, fully demonstrating its multimodal mechanosensing capability. As shown in Figure 5h, the relative resistance variation increases almost linearly with the torsion degree rising in both clockwise and counterclockwise directions, owing to the isotropic structure of the CGF‐Ecoflex hybrid sensing film.^[^ ^20^ ^]^ The unique structure also renders the device with symmetric sensing behaviors to convex and concave bending deformations (Figure 5i). A decrease in resistance was first observed upon bending; when the bending radius (*r*) was shortened to 10 mm, the resistance shifted to increase drastically with decreasing *r*. This can be well interpreted that slightly bending the film from flat status to the threshold *r* of 10 mm exerts a predominant pressure thus decreasing the resistance, whereas severely bending the film with *r* down to 4 mm exerts a predominant tensile strain thus increasing the resistance.^[^ ^16b^ ^]^

**Figure 6:**
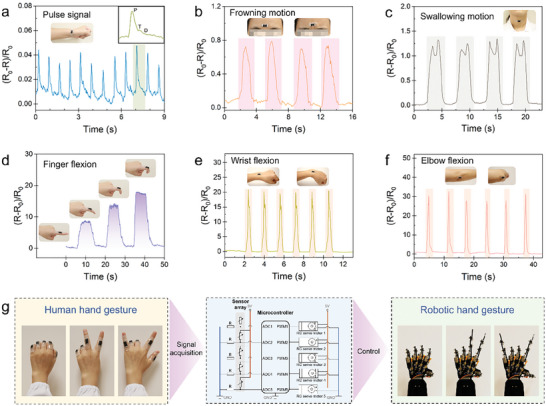
Full‐range human motion detection and application of the multimodal mechanosensors in HMI. a) Real‐time detection of wrist pulse. The insets show a photograph and a magnified pulse signal. b,c) Photographs and relative resistance changes of the sensor during subtle human motions: (b) frowning motion and (c) swallowing motion. d) Resistance signal in response to a human finger bending to different angles. Insets: optical images showing the finger under different bending angles. e,f) Optical images and resistance responses to large human motions: (e) wrist flexion and (f) elbow flexion. g) Schematic illustration of an intuitive human‐robot interaction, which realized the recognition of the hand gesture and the synchronous manipulation of the robot hand.

Owing to its softness, stretchability, lightweight, and conformability to human skin, the high‐performance CGF‐Ecoflex hybrid mechanosensor can function as an epidermal device to intimately monitor human physiological signals and motions. The sensor was conformably attached onto a human wrist to detect the real‐time pulse signal. **Figure** **6**a shows the recorded radial arterial pulse waveforms with three sub‐components of percussion (P), tidal (T), and diastolic (D) waves (inset); the radial augmentation index can be derived as A*I*_r_ = *T*/*P* to characterize the arterial stiffness thus providing important information for diagnosis of cardiovascular diseases.^[^ ^36^ ^]^ The epidermal mechanosensor was also placed on other parts of the body to acquire physical signals induced by human activities. For instance, when the sensor was mounted onto the middle of forehead and throat, it can recognize the frowning and swallowing motions as shown in Figure 6b,c. In addition to the subtle motions induced by muscle contraction, large motions associated with arthrosis movement can also be detected. Figure 6d shows the real‐time monitoring of finger flexion to different degrees by adhering the sensor to the index finger, while steady signals generated by fast wrist and elbow flexions were acquired in Figure 6e,f. The superior capability of full‐range human motion detection endows the epidermal mechanosensor with the potential for versatile HMIs. As a proof‐of‐concept demonstration, the soft and stretchable devices were directly attached to human fingers to implement an unobtrusive and comfortable HMI for sensorimotor prosthetic control of a robot hand. Figure 6g illustrates the algorithm in the control system that converts the input resistance variations of each mechanosensor on human fingers into output servo angles for different robot fingers. In a typical interaction process, the user sent a command by gesturing which generated resistance changes in the sensors. The resistance changes were collected and transformed into ascending/descending voltage values by the designed circuit, then were transferred into a microcontroller as the input analog signals. The microcontroller processed and converted the analog inputs to digital outputs and communicated with the robot hand through servo connection thus controlling the rotation angles of the corresponding servo motors. As a result, the robot fingers moved to a determined position so as to recapitulate the human hand gestures. As displayed in Figure 6g and Movie S1 (Supporting Information), various hand gestures can be accurately and synchronically reflected by the robot hand.

**Figure 7:**
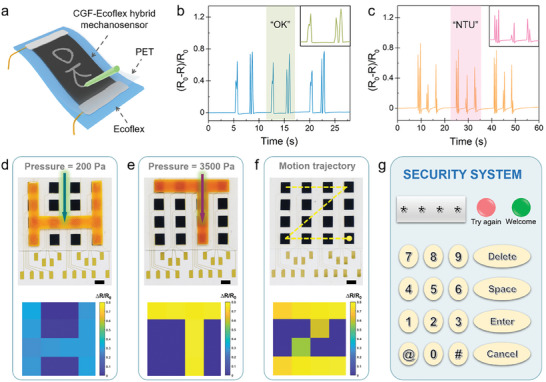
Advanced sensing applications of the CGF‐Ecoflex hybrid mechanosensor. a) Schematic diagram of a pencil writing on the sensor. The relative resistance changes in response to the signatures of b) “OK” and c) “NTU”. d,e) Photographs (top panel) and the corresponding pressure mapping (bottom panel) of the mechanosensor array pressed by an “H”‐ and “T”‐shape plate with different pressure levels. f) Schematic illustration of the movement of a human finger on the surface of the device (top panel) and the generated resistance signals from the 16 pixels (bottom panel). g) The representative graphical user interface (GUI) of a prototype security system.

The exotic merits of the multimodal mechanosensor, including ultrahigh sensitivity, low detection limit, wide detection range, and fast response behaviors, can prompt diverse advanced sensing applications. As exemplified in Figure S15 (Supporting Information), the sensor exhibited a rapid response with high consistency to subtle pressures induced by water droplets, suggesting its potential for precise perception of environmental feeble signals and human‐environment interactions. Intriguingly, the mechanosensor is also responsive to stimuli induced by movements on its surface. As demonstrated in **Figure** **7**a, a pencil was used to write letters on the CGF‐Ecoflex hybrid sensor surface which was covered by a thin polyethylene terephthalate (PET) layer to reduce the friction force during writing; the movement of pencil tip resulted in a structural distortion of the graphitic conductive network thus generating electrical responses. Handwriting of different words yielded distinct waveforms as compared in Figure 7b,c, which reflects this device is promising to be applied for smart signature recognition and anti‐counterfeiting. One of the stringent requirements of tactile sensing is to spatially resolve the distribution of external mechanical stimuli. As a prototype demonstration, a flexible mechanosensor array was facilely constructed through the “FsLDW‐elastomer infiltration‐physical transfer” process (Figure S16, Supporting Information). To explore its pressure mapping capability, a 3D‐printed “H”‐shape plate was first placed on the mechanosensor array, then a pressure of 200 Pa was applied on the plate (Figure 7d top panel). The corresponding relative resistance change (∆*R*/*R*_0_) of each pixel was measured and re‐constructed into a 2D contour map, which correlated well with the pressure profile (Figure 7d bottom panel). Similar result was obtained when a higher pressure of 3500 Pa was exerted on a “T”‐shape plate placed on the device (Figure 7e). These clearly demonstrate that the mechanosensor array can precisely differentiate the spatial distribution and the amplitude of a static pressure. In addition, the real‐time motion trajectory can be readily detected by the mechanosensor array. As shown in Figure 7f, an index finger moved on the surface of the array along a “Z”‐shape trace, the output resistance signal generated by each pixel successfully provided the motion path tracking information. Such an exceptional pressure mapping capability of the mechanosensor array paves the way for direct human‐machine communication; an exemplary application is to function as a touch panel in a security system, as demonstrated in Figure 7g and Movie S2 (Supporting Information). In the prototype security system, the mechanosensor array which enabled intuitive password entry was integrated with display and indicators. For any user to log in the security system, if the input password matched the default one, a green LED turned on, implying a successful authorization; otherwise, a red LED turned on, sending a warning message of the unsuccessful accessing process.

## 3 Conclusion

In conclusion, we have developed a high‐throughput manufacturing strategy to achieve epidermal multimodal mechanosensor arrays. Profiting from its high‐spatial‐resolution fabrication capability, the programmable UV FsLDW method allows for arbitrary patterning of thin‐layer CGF film with small pores. The CGF subsequently serves as the 3D framework for elastomer infiltration, resulting in a rigid CGF‐soft Ecoflex hybrid foam‐like thin film. The effective separation of interconnected graphitic flakes and the microcrack disconnection under tensile strain result in a sharp reduction in the conductive pathways. In the meanwhile, the soft Ecoflex filler maintains the structural integrity by absorbing the mechanical energy and continuously generating microcracks under increasing strain. Consequently, the CGF‐Ecoflex hybrid mechanosensor exhibits combined high strain sensitivity of crack‐based sensors and wide detection range of foam‐based sensors (GF of 371.4 in the first linear range of 0–50%, and maximum GF of 8922.6 in the sensing range of 61–70%), together with a low detection limit (0.01%), a rapid response/recovery behavior (2.6/2.1 ms), and excellent mechanical durability (<4% signal degradation in 4000 stretching‐releasing cycles). In addition, the mechanosensor demonstrates multimodal sensing capabilities to pressure, bending, and torsion. The outstanding softness and stretchability of the mechanosensor enabled its conformable attachment onto human body, so as to acquire a rich variety of physical and physiological signals in a high‐fidelity fashion. As proof‐of‐concept examples, a multi‐pixel mechanosensor array is facilely constructed through the simple “FsLDW‐elastomer infiltration‐physical transfer” process and successfully implemented in a security system and a robot controlling system, promising its applications in diverse intelligent HMI systems.

## 4 Experimental Section

### FsLDW Synthesis of CGF

Commercial PI sheets (Kapton tape, 3M, thickness of 0.076 mm) were selected as the precursor and graphitized into arbitrary patterns through a FsLDW process in ambient environment. A Yb‐doped fiber femtosecond laser (Lasernics, FUPL‐250‐6) with a third harmonic generated central wavelength of 347 nm, a pulse duration of 200 fs, and a repetition rate of 204.5 kHz was utilized as the laser source to construct the FsLDW platform. The CGF was synthesized with varying laser power from 100 to 600 mW at a fixed writing speed of 100 mm s^−1^.

### Fabrication of CGF‐Ecoflex Hybrid Mechanosensors

Biocompatible and skin‐safe silicone rubber (Ecoflex, Smooth‐On, 00–30) was made by mixing the two parts at a weight ratio of 1:1. After vacuum degassing for 10 min, the mixture was poured onto the as‐prepared CGF pattern (1 × 0.5 cm^2^ rectangle) in a mold. Curing the sample at room temperature for 2 h then peeling off the un‐converted PI layer enabled the full and consistent transfer of the CGF pattern onto the elastomeric substrate. Finally, the as‐produced CGF‐Ecoflex hybrid film was electrically wired to assemble the multimodal mechanosensor.

### Fabrication of Flexible Multi‐Pixel Sensing Matrix

The metal layer of Au/Ti (100/20 nm) with patterns of interdigitated electrodes and interconnects was deposited on a PET substrate by electron beam evaporation through a shadow mask. Large‐area patterning of 4 × 4 CGF array patterns (each pixel was 0.5 × 0.5 cm^2^ square) was achieved by a single‐step FsLDW on a large PI sheet. In analogy with single device transfer process, the CGF array patterns were transferred to a large piece of Ecoflex substrate, which was subsequently bonded to the PET substrate with the CGF‐Ecoflex hybrid film in direct contact with the Au electrodes (Figure S16a, Supporting Information). The as‐formed mechanosensor array was flexible and could be conformably attached to a human hand (Figure S16b, Supporting Information).

### Characterizations

The morphologies of as‐written CGF and as‐transferred CGF‐Ecoflex hybrid were revealed with a field‐emission SEM (JEOl, JSM‐7600F). Raman spectra were collected with a micro‐Raman spectroscope (Renishaw Invia) using a 633 nm laser line with a power of 3 mW. XPS data were acquired using a PHI‐5400 equipment with Al Kα beam source (250 W). The binding energies were calibrated using C1s peak (284.6 eV) as reference. The *R*_s_ was measured using a four‐point probe meter (Advanced Instrument Technology, CMT‐SR2000N). The tensile tests were carried out using a mechanical testing machine (Instron 5566) with a load cell of 100 N. The test coupons (Figure S17, Supporting Information) were pulled uniaxially at a speed of 500 mm min^−1^ until breaking.

### FEA Simulation

A finite element model was established using COMSOL Multiphysics 5.6 to examine the strain distribution of the mechanosensors under uniaxial stretching. The simulation was based on the steady state in the solid mechanics module, with following parameters: Young's modulus of 61 kPa, Poisson's ratio of 0.49, and density of 1.62 m^3^ kg^−1^. In the numerical model, one end of the CGF‐Ecoflex hybrid film was fixed while the other end was movable. The mesh was created through a physics‐controlled manner, the first principal strain was simulated by uniaxially stretching the movable end of the specimen.

### Electromechanical Measurements

Tensile strain and bending strain were exerted by clamping the device on a motorized linear translation stage (Thorlabs, DDSM100). A home‐built setup employing a motorized rotation stage (Thorlabs, DDR100) was used to induce torsional strain to the sensor. Static pressure sensing tests were conducted by gently loading different weights on the mechanosensor which was covered by a piece of PET sheet to uniformly distribute the pressure. The dynamic pressure was applied by a piezoelectric actuator (Thorlabs, PK25LA2P2) driven by a function generator (Rigol, DG4102). The amplitude and frequency of the applied pressure were controlled by those of the sine‐wave driving voltage. *I*–*V* and *I*–*t* characteristics were acquired using a source‐measurement unit (SMU, Keysight, B2902A).

The signal acquisition of the 4 × 4 sensing array was realized using an Arduino Mega board based on the ATmega2560 microcontroller which featured 16 analog inputs for reading the sensors. The resistance change of each pixel upon pressure was first transformed to a voltage change by a voltage divider circuit (in series connection with a 10 kΩ resistor). The output voltage signals were sequentially converted to digital values by the internal analog‐digital converter (ADC) module on the microcontroller. The collected data were sent to a computer for further analysis.

To function as a touch panel, the voltage signal of each pixel in the sensing matrix was monitored by the microcontroller and compared with its initial value. If there was a significant change in the read voltage of a channel, it was considered as a “button press” so that a yellow LED flashed to notify the user. In addition, the identification information was also received by the computer with the corresponding pixel highlighted on the GUI. To recognize the password input for accessing a security system, the sequence of the input key was compared with the stored number. A green LED was turned on if a correct sequence was received, with a “welcome” sign displayed on the GUI; otherwise, a red LED was triggered if the sequence was incorrect, and the GUI showed a notice for the user to try again.

To demonstrate the human‐robot interaction, five stretchable mechanosensors were wrapped around human fingers. Then every sensor was connected in series with a 10 kΩ resistor to construct a voltage divider. The resistance change of the sensor under human finger bending was converted to an output voltage as $V_{\mathrm{out}}=\;V\times \frac{R}{R+R_{\mathrm{sensor}}}$. The voltage signal flew into an Arduino Uno board embedded with a microcontroller (ATmega328P) and was converted to a digital value by the internal ADC module. Pulse with modulation was adopted to adjust the angle of the radio‐controlled servo motor attached to the robot hand, thus enabling the robot finger to bend or straighten.

The authors declare that written informed consent was obtained from all the participants prior to the enrollment of this study.

## Conflict of Interest

The authors declare no conflict of interest.

## Supporting information

Supporting Information

Supplemental Movie 1

Supplemental Movie 2

## Data Availability

The data that support the findings of this study are available from the corresponding author upon reasonable request.
