# Supplementary material for: High‐Throughput Manufacturing of Multimodal Epidermal Mechanosensors with Superior Detectability Enabled by a Continuous Microcracking Strategy
Source: Adv Sci (Weinh). 2023 Nov 30;11(4):2305777. doi: 10.1002/advs.202305777 (PMC10811494; doi:10.1002/advs.202305777)
Supplement: Supplementary file 1 — Supporting Information [file ADVS-11-2305777-s002.pdf]

ADVANCED SCIENCE
Open Access

Supporting Information

for *Adv. Sci.*, DOI 10.1002/advs.202305777

High-Throughput Manufacturing of Multimodal Epidermal Mechanosensors with Superior Detectability Enabled by a Continuous Microcracking Strategy

Jianing An, Van Thai Tran, Hai Xu, Wenshuai Ma, Xingkuan Chen, Truong-Son Dinh Le, Hejun Du, Gengzhi Sun* and Young-Jin Kim**

Supporting Information

High-Throughput Manufacturing of Multimodal Epidermal Mechanosensors with Superior Detectability Enabled by a Continuous Microcracking Strategy

Jianing An, Van Thai Tran, Hai Xu, Wenshuai Ma, Xingkuan Chen, Truong-Son Dinh Le, Hejun Du, Gengzhi Sun,* and Young-Jin Kim**

Prof. J. An, W. Ma
Institute of Photonics Technology, Jinan University, Guangzhou, 510632, P.R. China
E-mail: anjianing@jnu.edu.cn
Dr. V. T. Tran, Prof. H. Du
Singapore Centre for 3D Printing, Nanyang Technological University, 50 Nanyang Avenue, 639798, Singapore
H. Xu
College of Materials Science and Technology, Nanjing University of Aeronautics and Astronautics, Nanjing, 211100, P.R. China
Prof. X. Chen
Department of Chemistry, Jinan University, Guangzhou, 510632, P.R. China
T.-S. D. Le, Prof. Y.-J. Kim
Department of Mechanical Engineering, Korea Advanced Institute of Science and Technology (KAIST), Daejeon, 34141, Republic of Korea
E-mail: yj.kim@kaist.ac.kr
Prof. G. Sun
Institute of Advanced Materials (IAM), Nanjing Tech University (NanjingTech), Nanjing, 211816, P.R. China
E-mail: iamgzsun@njtech.edu.cn

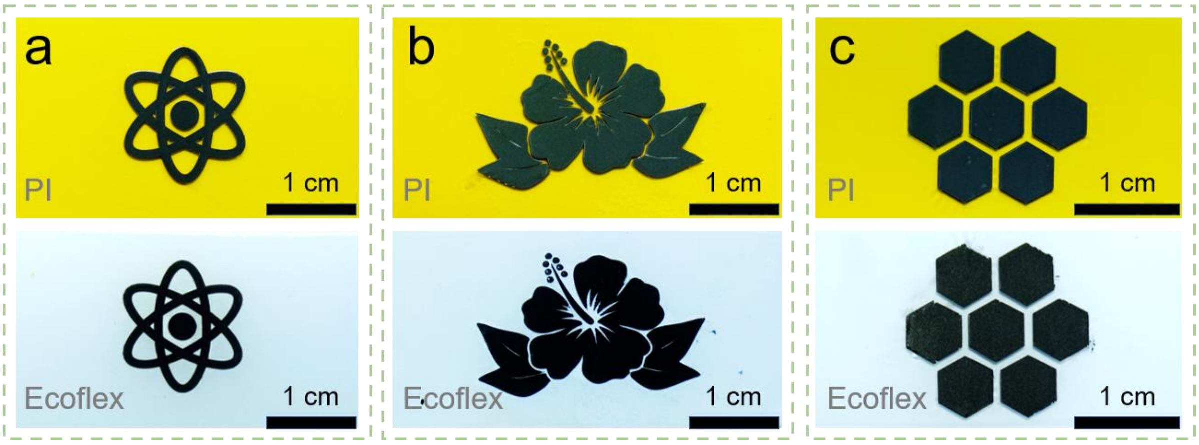

Figure S1. Femtosecond laser arbitrary patterning of CGF on PI (top panel) and the as-transferred patterns on Ecoflex (bottom panel).

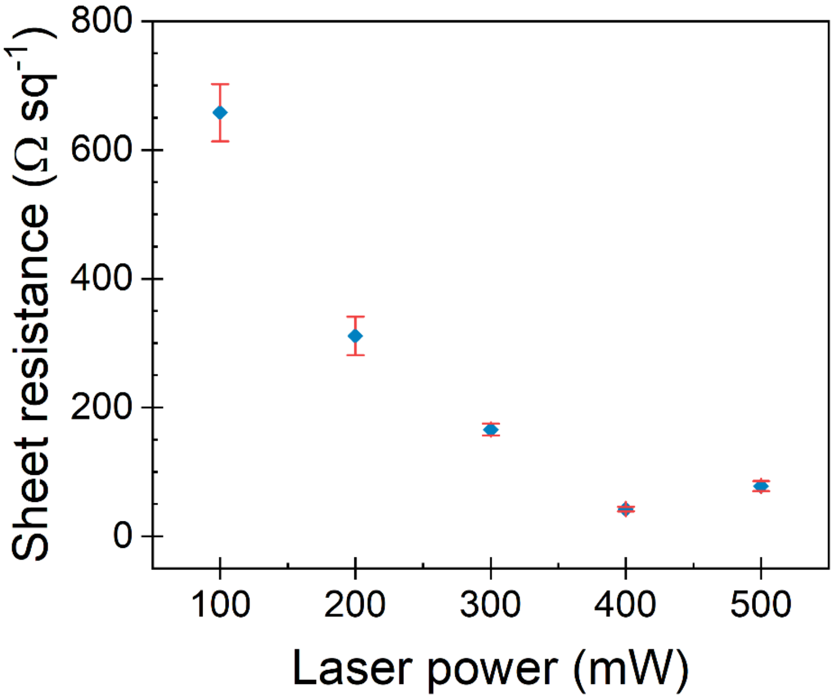

Figure S2. Plot of the sheet resistance of CGF film as a function of the laser power.

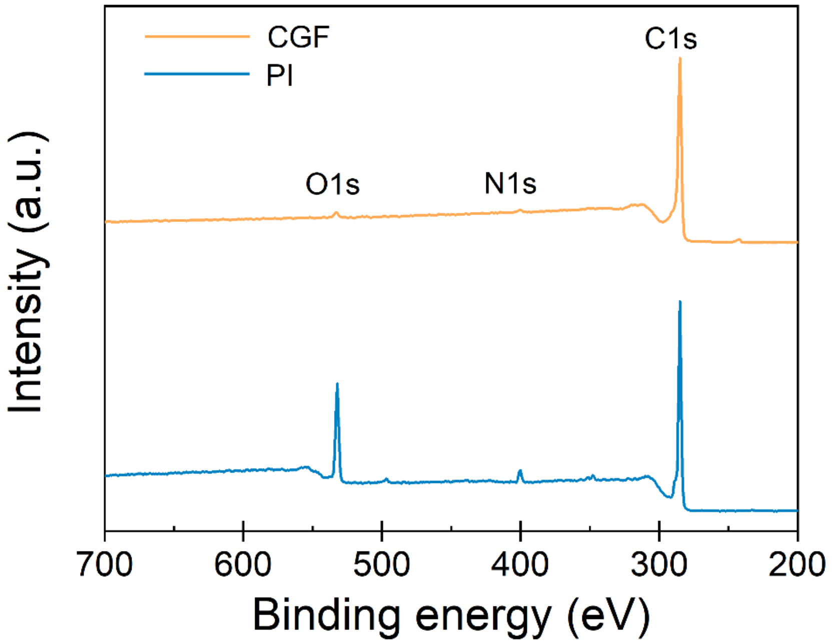

Figure S3. Broad scan XPS spectra of pristine PI and CGF.

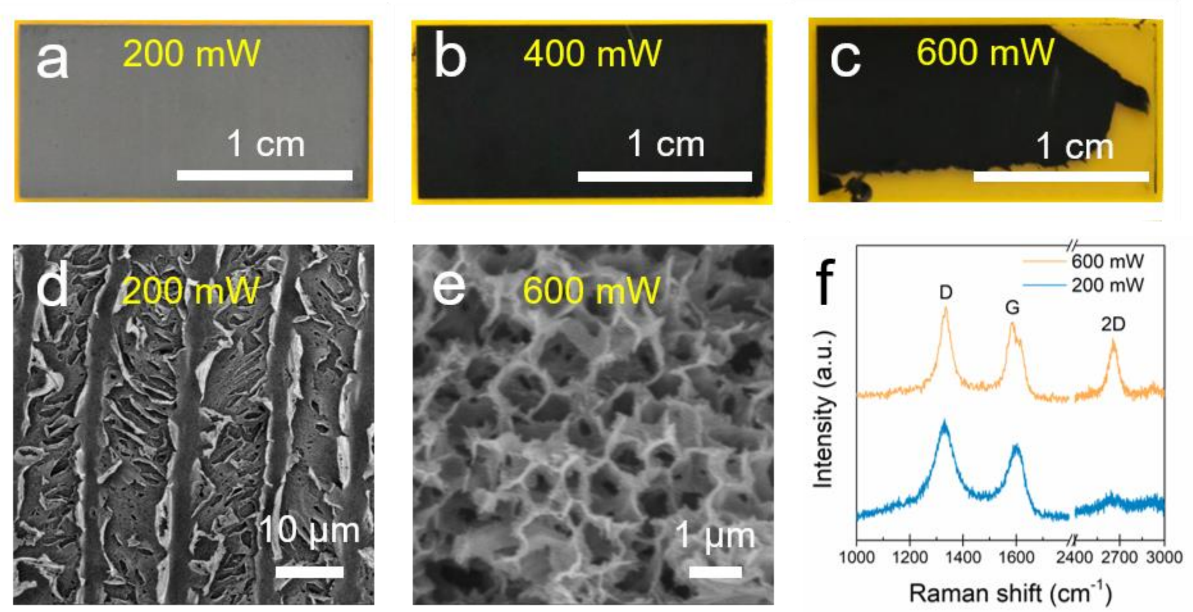

Figure S4. Optical images of as-written CGF films with a laser power of (a) 200 mW, (b) 400 mW, and (c) 600 mW. (d) SEM image showing sheet-like graphitic structures synthesized with a laser power of 200 mW. (e) SEM image showing porous graphitic structures written with a laser power of 600 mW. (f) Raman spectra of CGF produced with laser powers of 200 and 600 mW.

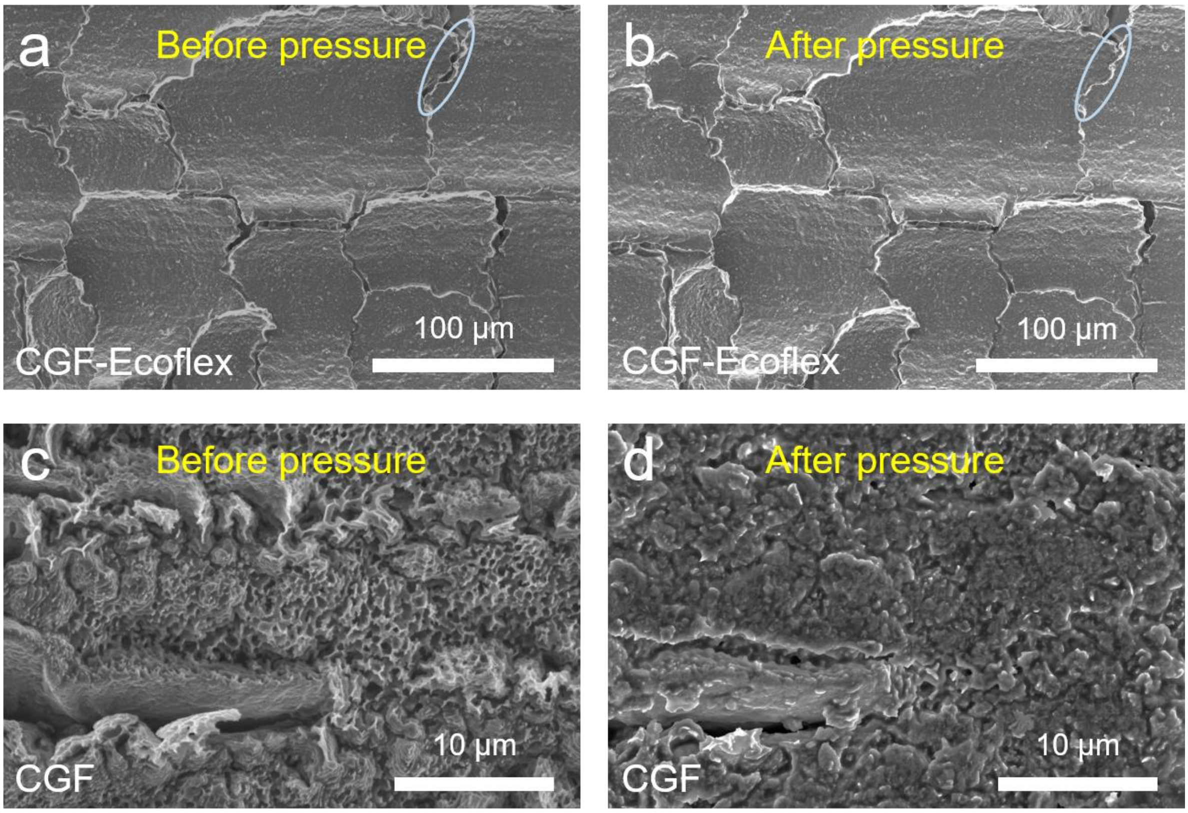

Figure S5. SEM images of CGF-Ecoflex and CGF before and after a compression test (compressive force = 50 N). The CGF-Ecoflex hybrid layer can retain its structural integrity under the compressive force, only microcrack closure was observed due to the compression (as circled in a and b). By contrast, the CGF layer collapsed during the compression test (c and d).

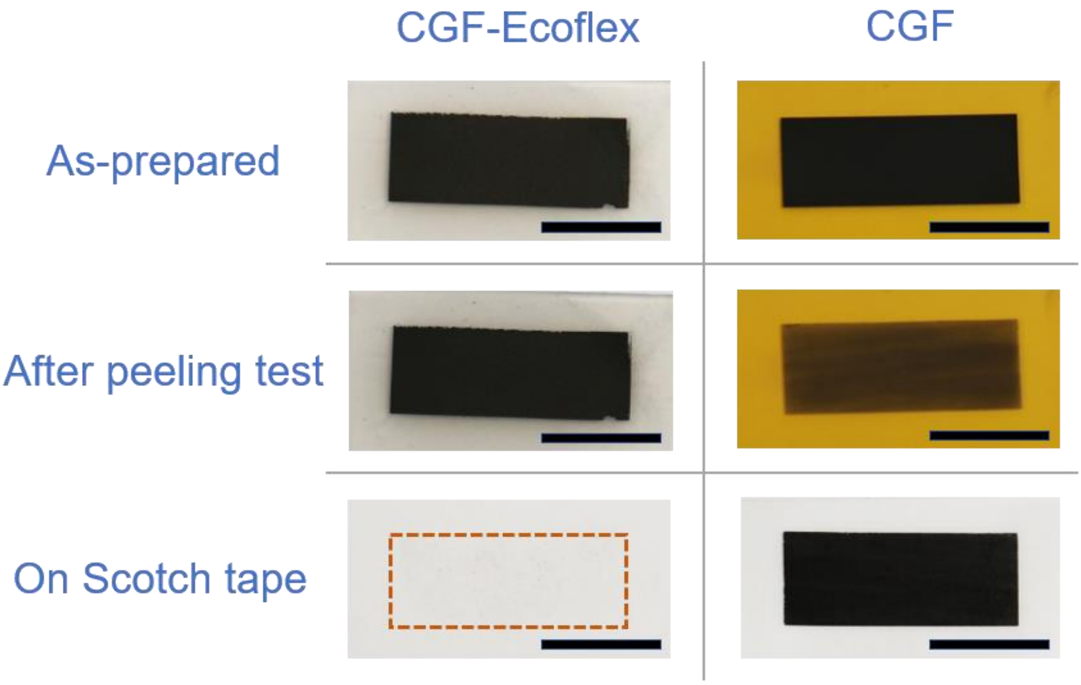

Figure S6. Adhesive tape peeling tests of CGF-Ecoflex (left column) and CGF (right column) samples. A Scotch tape was used to cover the film and the whole surface was pressed by a weight (10 g), then the tape was quickly peeled off. Top panel: photographs of the as prepared films. Middle panel: photographs of samples after adhesive tape peeling test. Bottom panel: photographs of the Scotch tape after peeling test. Negligible amount of material can be observed from the Scotch tape (marked by the red rectangle) peeled off from the CGF-Ecoflex film, indicating its excellent mechanical robustness. As a comparison, a large amount of material was transferred to the Scotch tape for CGF on PI. Scale bars: 10 mm.

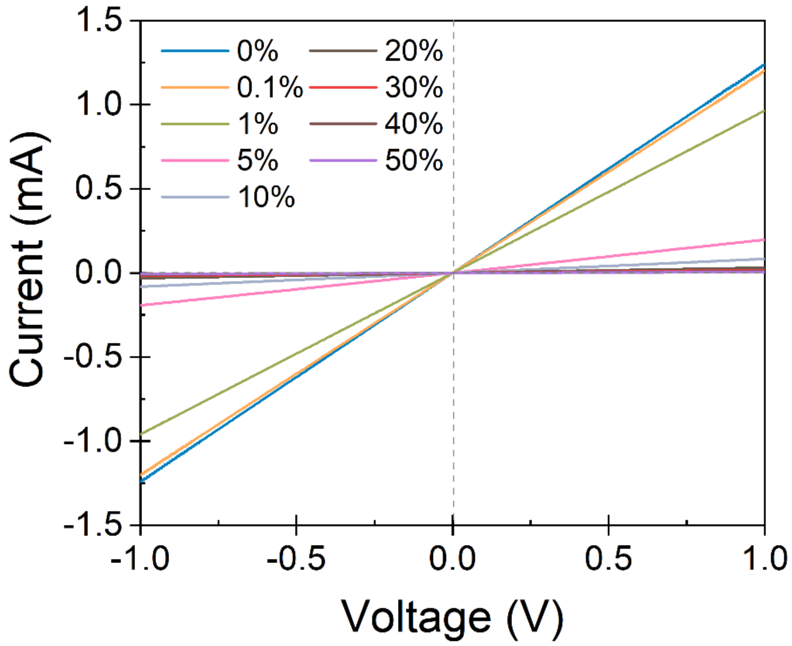

Figure S7. *I-V* curves of the mechanosensor under different tensile strains.

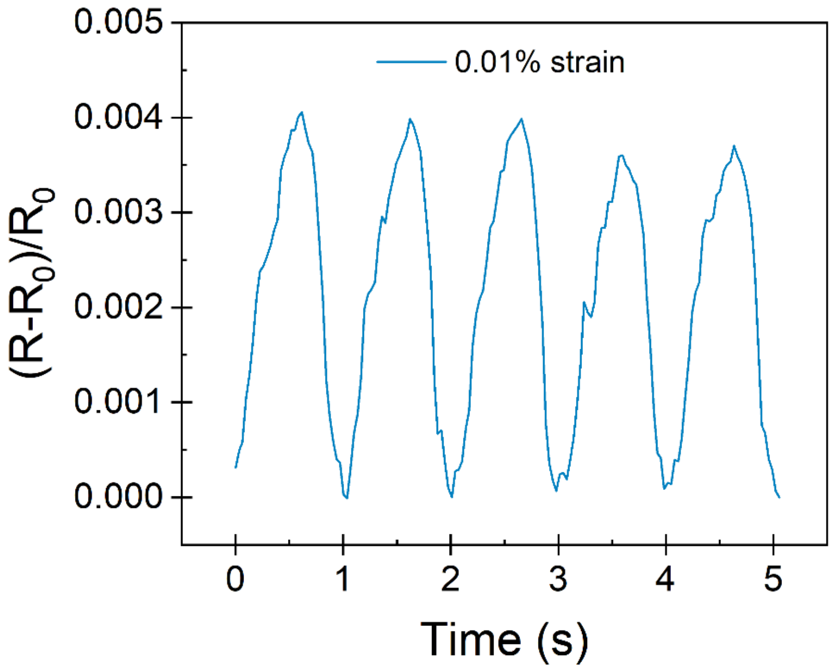

Figure S8. Dynamic response of the mechanosensor to a subtle strain of 0.01%. The minimum incremental movement of the translation stage is 5 μm. To achieve 0.01% strain, the clamping length of the sample is set to 5 cm. The sweep rate is 0.01 mm s^{-1}.

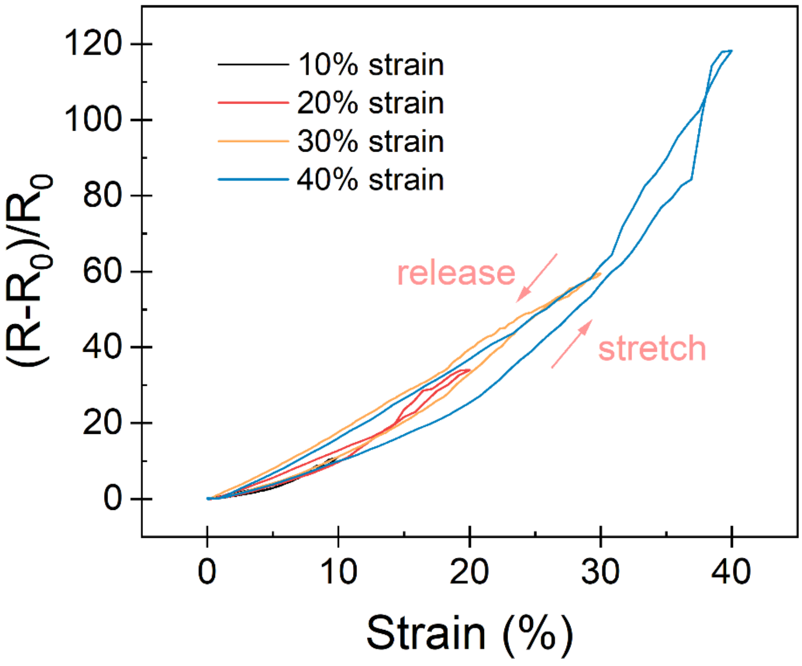

Figure S9. Hysteresis effects of the CGF-Ecoflex hybrid mechanosensor at different strains.

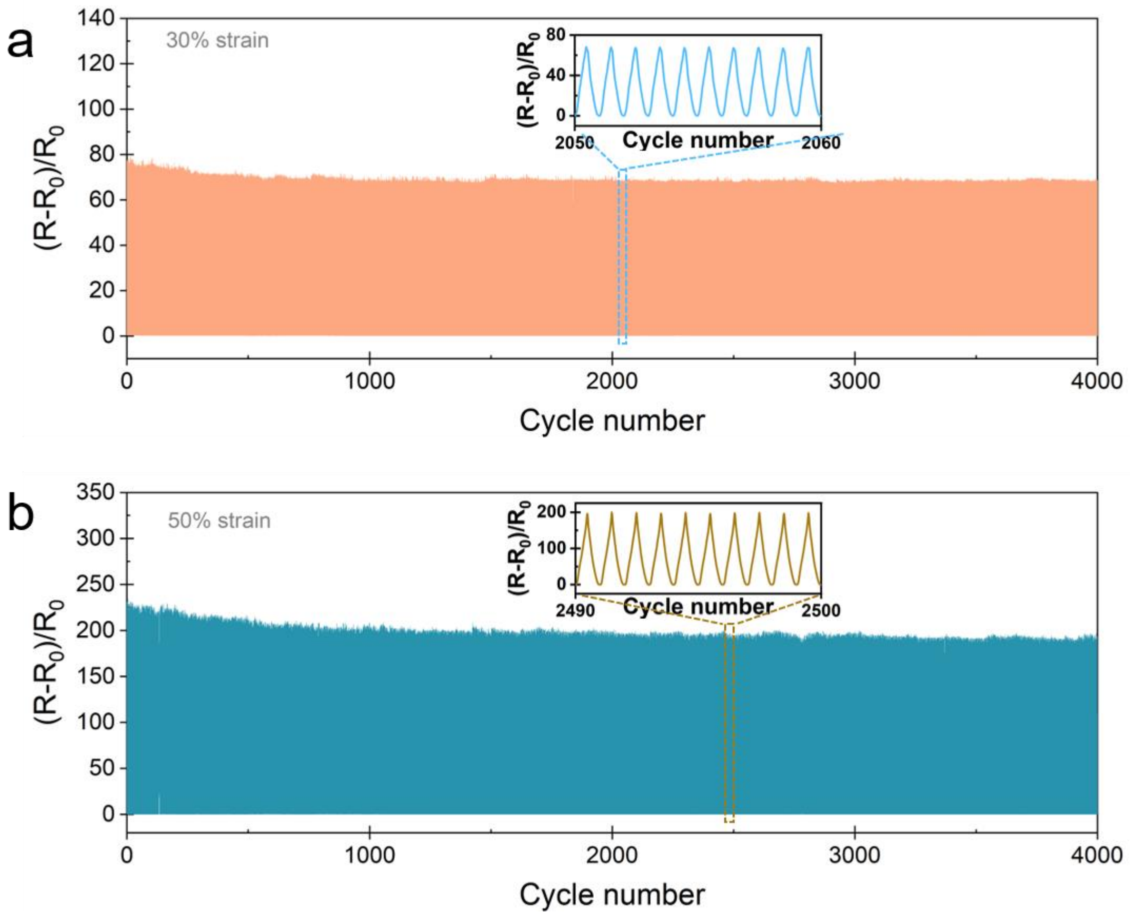

Figure S10. Durability tests of the mechanosensor under a tensile strain of (a) 30% and (b) 50%, respectively.

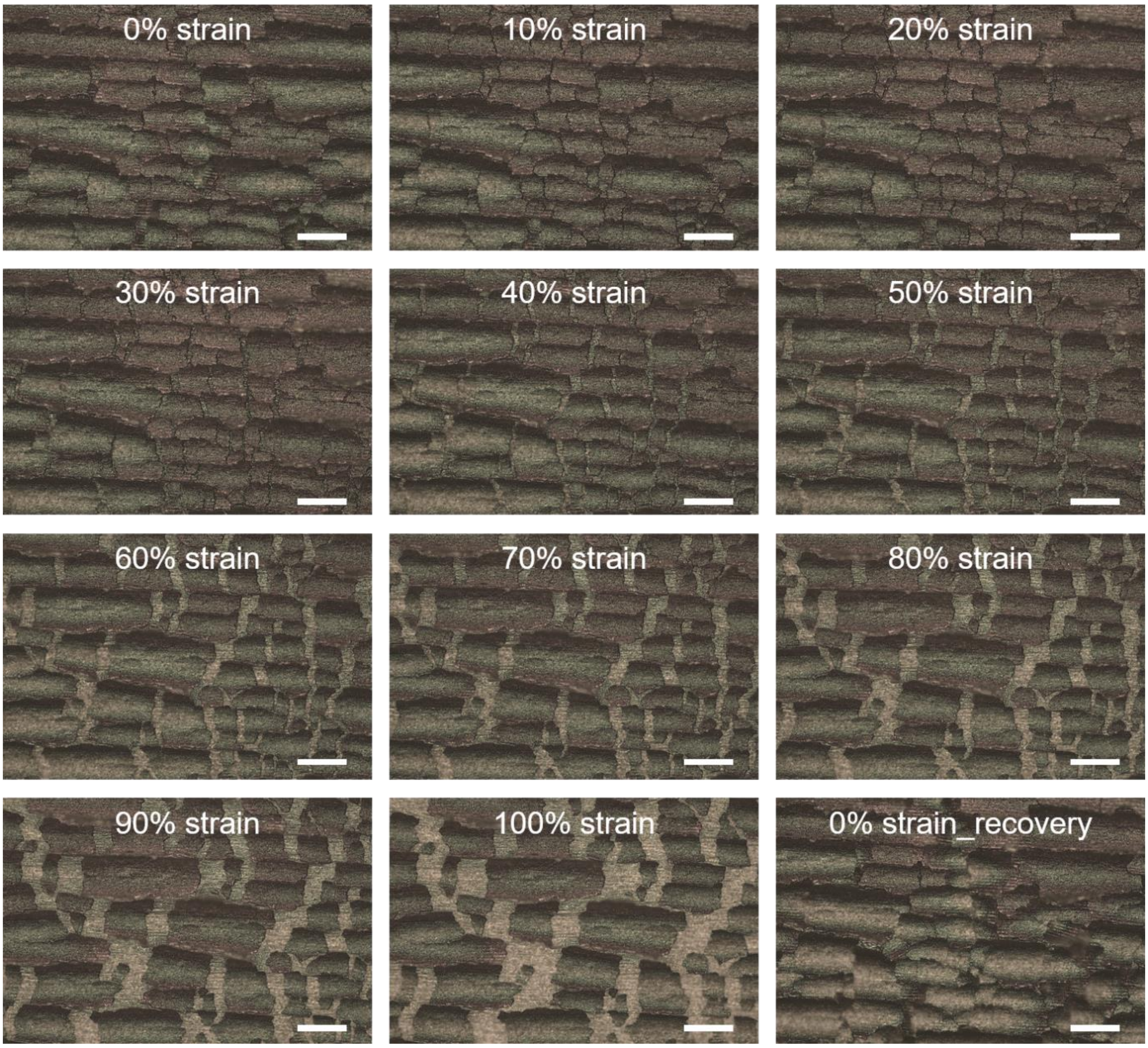

Figure S11. In-situ optical images of microcrack disconnection under different tensile strains (0-100%) and recovery to original state. Scale bars: 100 μm.

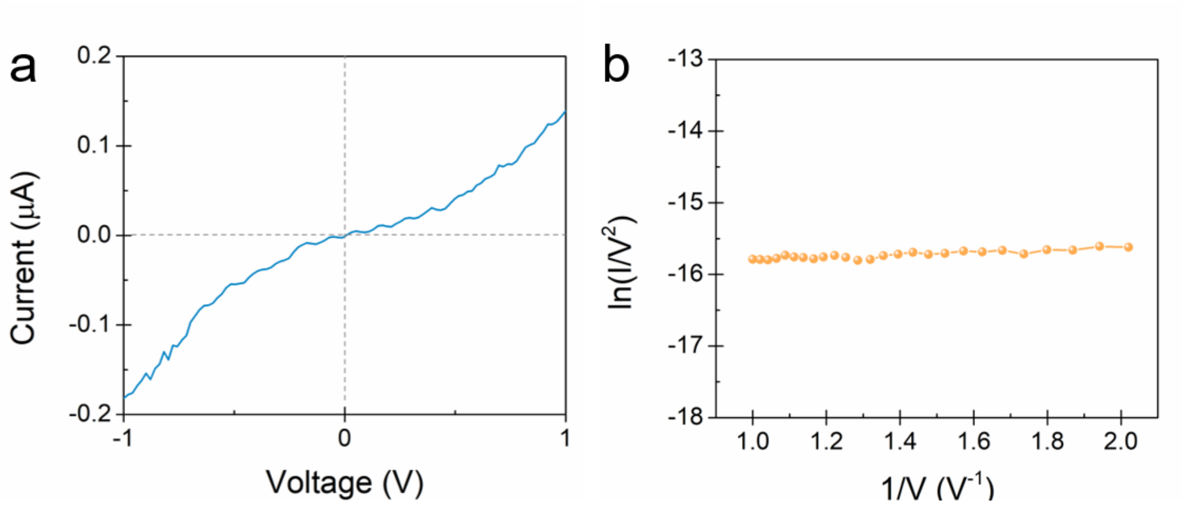

Figure S12. (a) *I*-*V* curve of the sensor under a large strain of 70%. (b) Plot of ln (I/V^2) versus 1/*V* in the voltage range of 0.5 to 1 V for the sensor under 70% tensile strain, the good linear behavior indicates the electron conduction follows a Fowler-Nordheim tunneling mechanism.

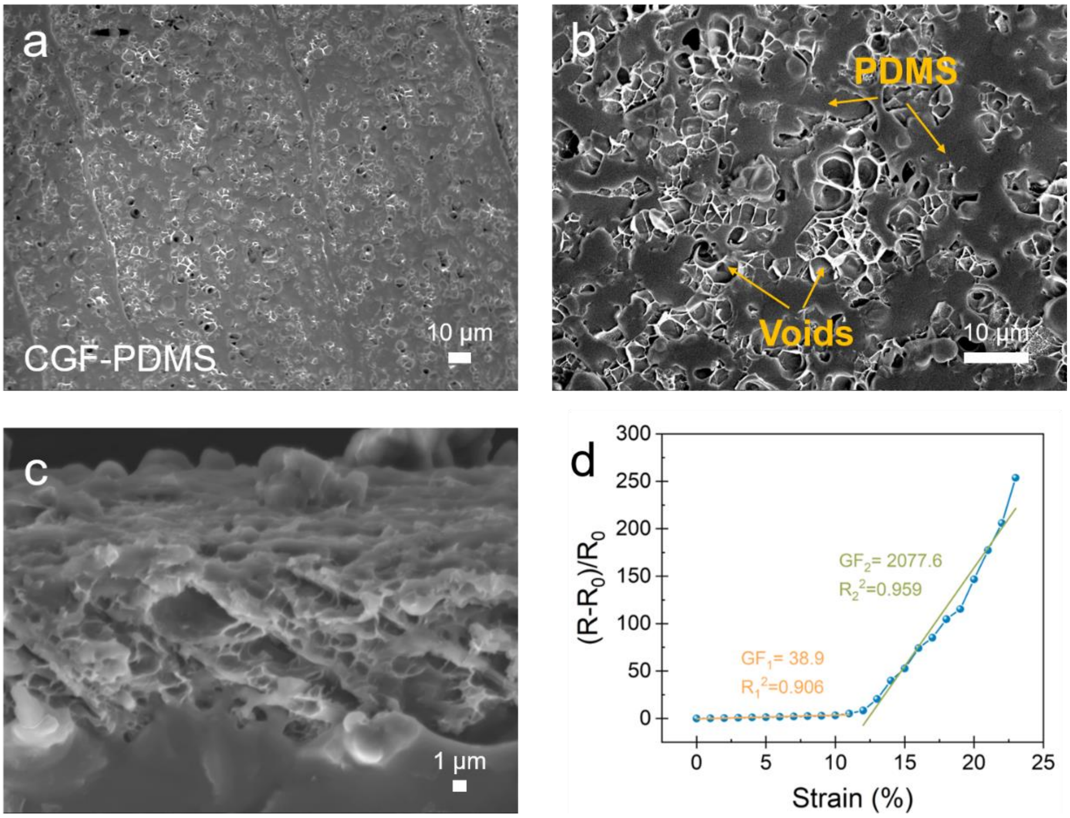

Figure S13. (a) SEM, (b) zoom-in view and (c) cross-sectional view of CGF-PDMS hybrid structure. Plenty of voids can be observed, indicating the PDMS is not fully embedded within the CGF framework. (d) Relative resistance variation-strain curve of the CGF-PDMS hybrid sensor.

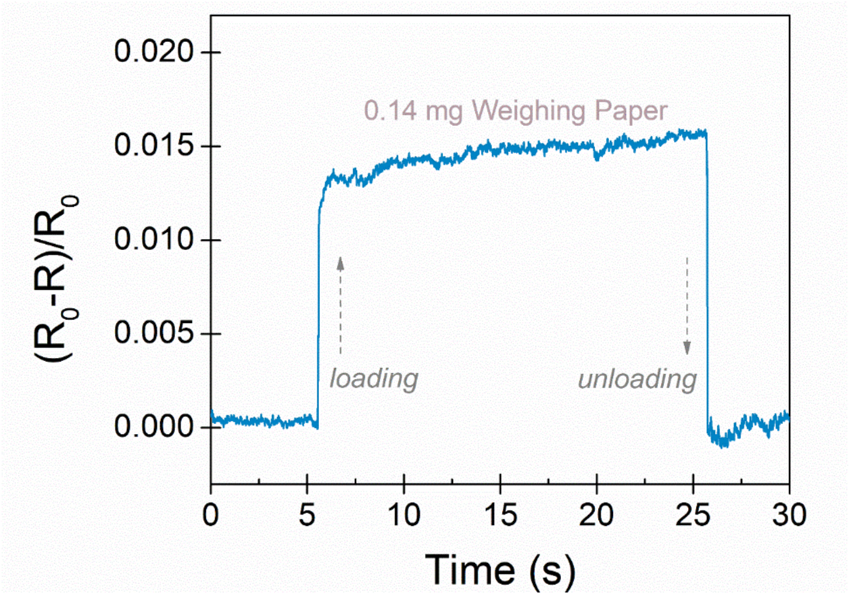

Figure S14. Real-time response of the CGF-Ecoflex hybrid mechanosensor to a subtle pressure induced by a small piece of weighing paper (0.14 mg).

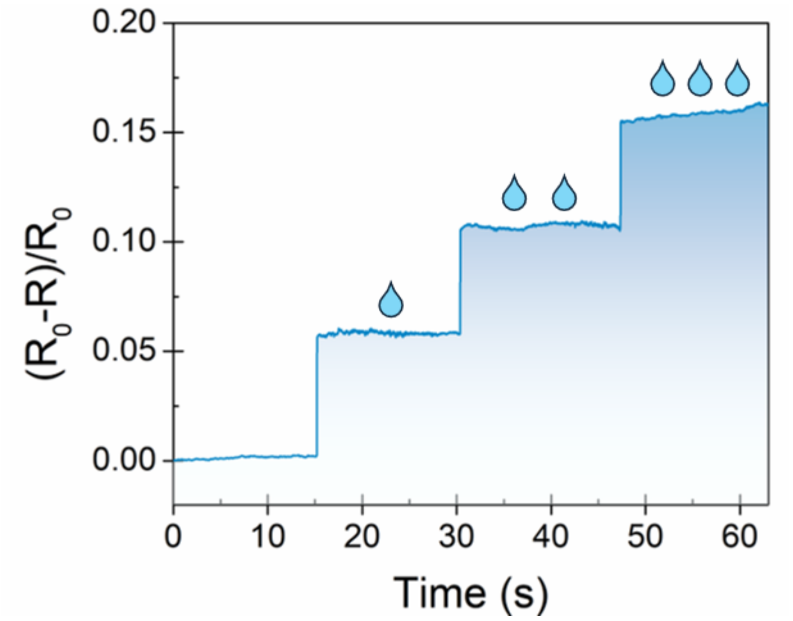

Figure S15. Real-time response to pressures induced by the applied water droplets.

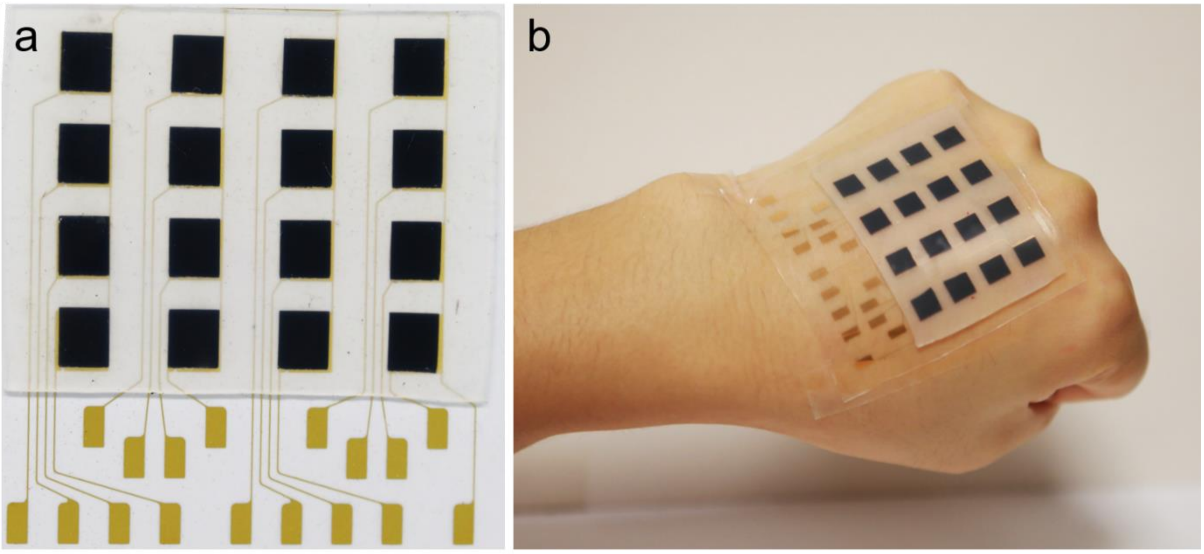

Figure S16. (a) Photograph of a flexible mechanosensor array composed of Au electrodes and CGF-Ecoflex hybrid sensing film, the dimension of each pixel is 0.5 × 0.5 cm^2. (b) The mechanosensor array can be conformably attached onto a human hand.

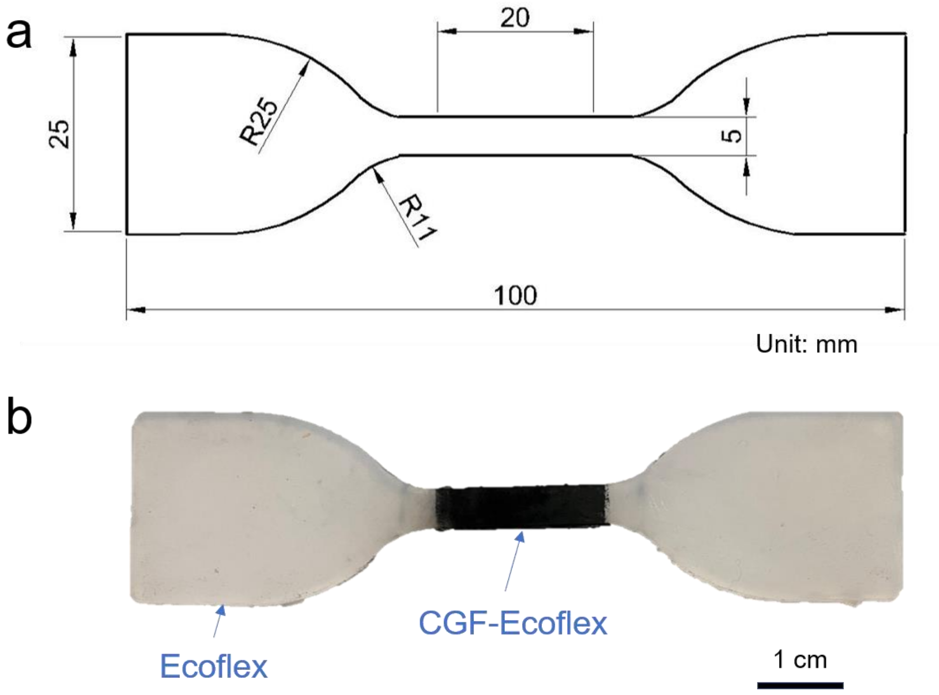

Figure S17. (a) Diagram and (b) optical image of the tensile test coupon.
